# Prevalence of Sleepwalking: A Systematic Review and Meta-Analysis

**DOI:** 10.1371/journal.pone.0164769

**Published:** 2016-11-10

**Authors:** Helen M. Stallman, Mark Kohler

**Affiliations:** Centre for Sleep Research, University of South Australia, Adelaide, South Australia, 5001, Australia; Universidade Nova de Lisboa Instituto de Higiene e Medicina Tropical, PORTUGAL

## Abstract

Sleepwalking is thought to be a common arousal disorder; however, the epidemiology of this disorder has not yet been systematically examined. A systematic search of MEDLINE, CINAHL, EMBASE, PsycINFO, PubMed, and ScienceDirect was conducted for ‘sleepwalking’ OR ‘somnambulism’ in any field, to identify studies that reported the epidemiology of sleepwalking or sleepwalking disorders. Fifty-one studies assessed the prevalence rates of sleepwalking in a total sample of 100 490. The meta-analysis showed the estimated lifetime prevalence of sleepwalking was 6.9% (95% CI 4.6%–10.3%). The current prevalence rate of sleepwalking—within the last 12 months—was significantly higher in children 5.0% (95% CI 3.8%–6.5%) than adults 1.5% (95% CI 1.0%–2.3%). There was no evidence of developmental trends in sleepwalking across childhood. The significant risk of bias across all studies suggests these results should be used cautiously. Further epidemiological research that addresses methodological problems found in studies of sleepwalking to date is needed.

## Introduction

Sleepwalking is a behavior characterized by partial arousal during slow wave sleep (N4) [1]. The potential adverse health outcomes of sleepwalking are injury to the sleepwalker themselves or to others as a result of impaired perception, characteristic of sleepwalking. The most sensationalized of these adverse events come to the public’s attention (e.g.[2]), otherwise sleepwalking largely goes unnoticed and may not get routinely reported to any health service. An absence of sleepwalking being recorded as a cause of significant injury requiring hospitalization or death (e.g.[3, 4–6]) may be: 1) indicative of very low prevalence rates of sleepwalking; 2) a reflection of the low rates of adverse events from sleepwalking; and/or 3) represent inadequate identification, reporting, or assessment of sleepwalking as the cause of injuries. Understanding the epidemiology of sleepwalking is important to public health, individual decision-making and clinical management. It can inform optimal allocation of health resources for this largely neglected behavior. General population screening is needed to understand the potential health implications [7].

### Challenges in epidemiological research for sleepwalking

The definition of sleepwalking varies considerably within the literature. The behavioral event is similar to the proverbial tree falling in the forest—if it is not observed, did it make a noise? Studies of children frequently rely on observation, typically using parent-report that their child sleepwalks, as the operationalization of sleepwalking. This reduces prevalence rates to those where the child captures the parents attention (e.g. such as by leaving their bedroom), are observed by parents, and the episode is later recalled by the parent. Some studies with older children use self-report, as do studies with adults. These are used to obtain lifetime and point prevalence rates, despite amnesia for the event being a common feature of the behavior. The classification of sleepwalking as a disorder rather than just a behavior, requires recurrent episodes, contact with others during the event, and amnesia for the event [8]. The American Psychiatric Association classifies sleepwalking as a mental illness if, in addition to the ICD-10 CM [8] characteristics, the events cause clinically significant distress or impairment in social, occupational or other important areas of functioning [9]. The increasing complexity of the definitions would be expected to result in decreasing prevalence rates, with sleepwalking behavior being the more prevalent and the mental illness of sleepwalking least likely to occur.

These differing levels of operationalizing sleepwalking necessarily result in different measurement strategies. Polysomnography (PSG) is the only measure that can accurately confirm the neurological event of sleepwalking—demonstrated by ambulant behavior during a maintained sleep state. However, PSG can be impractical to do on a large scale and may miss sleepwalking episodes that are usually infrequent. Fallible measures of sleepwalking include actigraphy, video monitoring, direct observation, self-report, and significant other report. Actigraphy is sensitive in detecting unique sleep patterns associated with specific sleep disorders [10]. It can provide an objective measure of sleep fragmentation due to movement, as a proxy measure of nocturnal wandering. Immediate parent-report relies on the child being observable to parents. Self-report relies on at least partial awareness of the event by the individual, or being told about their sleepwalking by someone who has observed it. Given that amnesia is a common feature of sleepwalking, sleepwalkers who are observed (e.g. children) would be more likely to be aware of sleepwalking than those who live alone. This most likely explains higher rates of sleepwalking in adults who are married compared with those who are single [11].

Retrospective recall is reliant on encoding the event as significant and long-term recall of the episode [12]. Distinctively different sleepwalking experiences would be more likely to be remembered by both sleepwalkers and their family members [13]. The distinctiveness of the episode constrains processing at the time of recall and thus reduces the incidence of false recall [14]. The measurement of the incidence of sleepwalking is likely to be more accurate than period prevalence, as a new experience of sleepwalking would be more distinctive to both parents/significant others and sleepwalkers than historical occurrences. Self-report could be based on memory for the event, distinctive features of an event such as injury or waking somewhere unusual, or reliant on what others have told the sleepwalker—each contains inherent measurement error. There is also the potential that individuals and observers could incorrectly classify all nocturnal wandering as sleepwalking.

Partially validated datasets are recommended in cases where the outcome variable is difficult or costly to measure [15], as in the case of sleepwalking. This involves all data points being classified by fallible tests and some of the data points being validated by also being classified by an accurate gold-standard test. To accurately assess the prevalence of sleepwalking, this would involve all participants being classified by fallible tests (such as self-, and parent-reports, actigraphy, or video monitoring) and some data points being validated by polysomnography. A partially validated dataset enables the systematic error that is included in each fallible measure to be quantified and taken into account in determining the true prevalence of sleepwalking.

Prevalence rates of sleepwalking frequented quoted in the literature typically relate to a single study (e.g. [16] or provide no reference at all e.g. [17, 18]). The operationalization of sleepwalking is rarely mentioned. By combining studies, taking into account the differing conceptualization and measurement of sleepwalking and assigning the individual studies different weights according to their sample size, the potentially troublesome role of individual studies is minimized. The aim of this study is to systematically examine the epidemiology of sleepwalking in general population samples of children and adults.

## Method

This study was registered with PROSPERO (#CRD42016036296). PRISMA guidelines were followed in conducting and reporting the results of this systematic review and meta-analysis [19].

### Search strategy

The following databases were included in the identification of relevant studies: MEDLINE, CINAHL, EMBASE, PsycINFO, PubMed, and ScienceDirect. Search terms were ‘sleepwalking’ OR ‘somnambulism’ in any field (e.g. PubMed search terms (sleepwalk*) OR somnambulism). The combined lists were screened for relevant titles and abstracts and full texts of all potentially relevant titles were examined. Studies were included if: 1) they reported the prevalence or incidence of sleepwalking; and 2) they were submitted to a peer-reviewed publication. All ages were included. The search was conducted in English; however, studies identified in other languages were included. Studies were excluded if: a) sleepwalking incidence or prevalence was not reported separately from other sleep disorders; b) participants were forensic cases or sleep-clinic samples; c) the study included drug-induced sleepwalking; or d) adults participants were psychiatric patients. Adult psychiatric patients were excluded because sleepwalking has been identified as a potential side-effect of psychotropic medications [20]. We identified other pertinent studies through citation tracking, review of reference lists in retrieved articles, Google Scholar, and our knowledge of the literature. The searches were from the beginning of each database through to 15 March 2016. All initial searches were conducted by the first author. The articles were then independently examined by the second author—there were no disagreements between authors.

### Data extraction

Data were extracted independently by both authors. For each paper, we documented authors, year, country, study design (e.g. cross-sectional, longitudinal), setting (e.g. school, general population), participants (e.g. adults, children), sample size, response rate, age range, data collection procedure, sleepwalking measure, and results.

### Data evaluation

We applied published guidelines for evaluating prevalence studies [21], using eight critical appraisal criteria across three domains, sampling, measurement, and data analysis. These are consistent with STROBE guidelines for the reporting observational studies in epidemiology [22]. Sampling items assessed whether the survey design yielded a sample of respondents’ representative of a defined target population. The items were whether: 1) the target population was clearly defined; 2) probability sampling was used to identify potential respondents; and 3) the characteristics of the respondents matched the target population. Measurement items assessed whether survey instruments yielded reliable and valid measures of sleepwalking. The items were whether: 4) the data collection methods were standardized; 5) the instruments were reliable; and 6) the instruments were valid. Data analysis assessed whether special features of the sampling design were accounted for in the analysis (Criterion 7). Confidence intervals, essential to produce frequency estimates within the population overall, were calculated for each study (Criterion 8). Evaluations was conducted independently by each author.

### Analyses

The prevalence of sleepwalking was calculated for each study with the number of reported sleepwalkers in the sample as the numerator and the total sample size as the denominator. All rates were calculated as the rate of sleepwalkers per 100 people, with the total sample being the summation of sleepwalker and non-sleepwalkers. An aggregate effect size, weighted by sample size, was computed to provide an overall effect size across the studies to identify the lifetime and current prevalence rates in children and adults. A random-effects model was used to aggregate individual effect sizes to create a pooled prevalence of sleepwalking. Random-effects models are based on the assumption that the true effect could vary between studies [23].

Homogeneity across studies was tested with the *I*^2^ index, which provides the percentage of variation in prevalence attributable to between-study heterogeneity. An *I*^2^ value of >75% is interpreted as high heterogeneity [24]. Post-hoc sensitivity analyses were conducted for the different study populations (child and adult) and measures (sleepwalking behavior and sleepwalking diagnoses) to investigate possible sources of heterogeneity. A forest plot was created to illustrate the prevalence of each study or current and lifetime sleepwalking, with 95% confidence intervals that contributed to the analysis along with the pooled prevalence estimate. Meta-regression was used to identify any developmental trends in current sleepwalking rates across childhood. Funnel plots [25] and Egger’s test of asymmetry [26] were used to formally detect bias within the results. All analyses were performed with Comprehensive Meta-Analysis Version 3 [27].

### Description of studies

**Fig 1:**
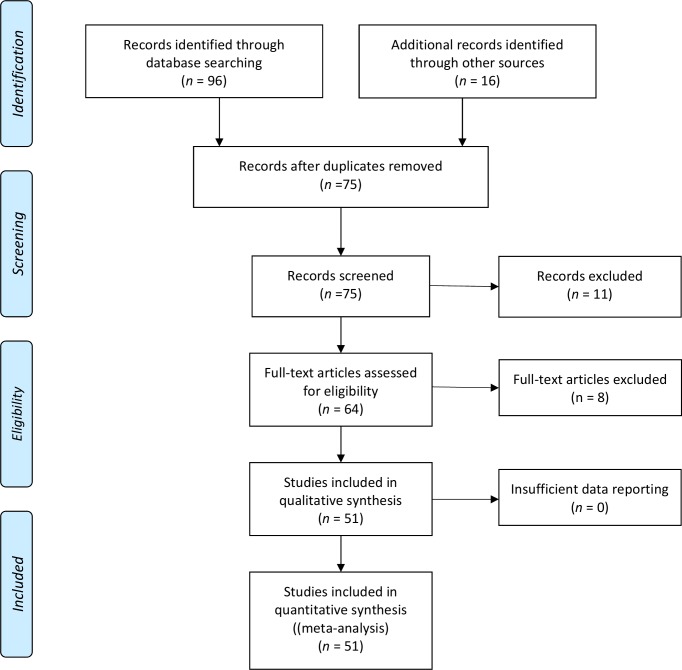
PRISMA flow of information through the different phases of the systematic review.

A total of 801 hits were produced in the database search. Review of titles resulted in 96 potentially relevant papers that were reviewed. Fig 1 displays the flow of information through the different phases of the systematic review and meta-analysis. From the original 64 sourced papers, 56 papers describing 51 studies met the inclusion criteria and were subsequently included for review. The eight full-text papers that were excluded were for the following reasons: not an empirical study (*n* = 1), not a general population sample (*n* = 5), not measuring sleepwalking epidemiology (*n* = 1), and not reporting sleepwalking separately from other sleep problems (*n* = 1).

A summary of the participant characteristics from the included studies is shown in Tables 1 and 2. They span more than seven decades of research and include 20 countries—representing an international and cross-cultural sample. There were 15 studies of adults comprising 31 108 participants and 36 pediatric samples comprising 69 382 children. Sample sizes ranged from 100 to 15 929 participants. All used an observational study design using questionnaires or interviews. The majority of studies measured sleepwalking behavior (*n* = 43). Eight studies measured sleepwalking using a diagnosis—five using DSM-IV [28] and three ICSD [29]. Several studies reported assessing ICD [30] and DSM diagnoses; however, as DSM has an additional criterion compared with ICD, that is ‘episodes cause clinically significant distress or impairment’ ([9]; p. 399) and only one result is reported in all studies, it is assumed the result refers to DSM. With child samples, six used child self-report and 26 used parent-report of sleepwalking behavior. Where children were asked to complete questionnaires with their parents, the results are included as parent-report.

**Table 1:** Characteristics of Included Studies of Children listed by Measurement Type.

Citation	Country	Design	Age Range	*N*	Response Rate %	Prevalence Outcomes	Criteria	Results %
**Self-reported behavior**								
Abdel-Khalek [37]	Kuwait	Cross-sectional	14–18	2574	ns	1 month	‘much’ and ‘very much’ response on 5-point scale (no, a little, moderate, much, very much)	10.0
Ghalebandi et al [38]	Iran	Cross-sectional	5–10+	4309	71.82	ns	‘almost always’ and ‘frequently’ response on 5-point scale (almost always, frequently, occasionally, rarely, never)	0.39
Ipsiroglu et al [39]	Austria	Cross-sectional	10–15	332	99.70	Lifetime	‘very often’ and ‘occasionally’ on 3-point scale (never, occasionally, very often)	15.10
Stallman et al [40]	Australia	Cross-sectional	17–18	532	ns	1 month	How frequently in previous month not during the past month’, ‘less than once a week’, ‘once or twice a week’, ‘three or more times a week’	2.90
Wiechers et al [31]	Germany	Cross-sectional	*M* = 9.6	1144	65.00	Current	‘sometimes’ or ‘often’ on 3-point scale (never/rarely, sometimes, often)	4.80
Yang et al [41]	China	Cross-sectional	12–18	846	ns	6 months	At least one experience	4.00
**Parent-reported behavior**								
Abe [42]	Japan	Cross-sectional	3	342	55.97	Lifetime	Yes/no	4.58
Abe et al [43]	Japan	Longitudinal	8	363	50.14	Lifetime	ns	4.13
Agargun et al [44]	Turkey	Cross-sectional	7–11	971	86.60	6 months	‘frequently/always’ response on 4-point scale (never, rarely, occasionally, frequently/always)	1.2
Archbold et al [45]	USA	Cross-sectional	2–14	1038	74.14	Lifetime	Yes/no	14.8
Bharti et al [46]	India	Cross-sectional	3–10	103	ns	ns	ns	1.90
Blader et al [47]	USA	Cross-sectional	5–12	987	59.82	6 months	Any response greater than ‘none’ on 4-point scale (none, <1 night per month, 1–2 nights per week, >3 nights per week)	9.80
Buhler & Largo [48]	Switzerland	Cross-sectional	6–18	320	ns	Lifetime	ns	5.00
Cai et al [49]	China	Cross-sectional	2–12	3756	ns	Lifetime	ns	0.90
Fisher & Wilson [50]	Canada	Cross-sectional	5–18	1695	40.03	12 months	“At least once”	21.00
						Current	“Still sleepwalking”	14.00
Goodwin et al [51] ^a^ Furet et al [52]	USA	Longitudinal	6–11	480	6.80	Current	more than three times/month on 4-point scale (Never, less than three times per month, three to five times per month, or more than five times per month)	3.50
			9–17	350	4.96	1 month		1.40
Greene et al [53]	UK	Longitudinal	5	7830	45.53	Lifetime	‘mild’ and ‘severe’ response on 3-point scale (non, mild, severe)	24.19
Kilincaslan et al [32]	Turkey	Cross-sectional	*M* = 16.09	3485	92.20	6 months	Above median number of occurrences in cohort	5.70
Klackenberg [54–56]	Sweden	Longitudinal	8 16	194 180	97.34 85.00	Lifetime	‘seldom’ response or more on 5-point scale (never, seldom, sometimes, often, always)	6.29 40.0
Laberge [57]	Canada	Longitudinal	13	1353	67.65	Lifetime	Presence of sleepwalking	5.80
Lehmkuhl et al [58]	Germany	Cross-sectional	*M* = 5.52	4793	28.9653.30	Current	‘sometimes’ or ‘often’ response on 3-point scale (never, sometimes, often)	3.3
Liu et al [59]	China	Cross-sectional	7–13	517	91.50	1 week	Yes/no	6.00
Liu et al [60]	China	Cross-sectional	2–12	5979	90.59	6 months	Yes/no	0.60
Neveus et al [33]	Sweden	Cross-sectional	6–11	1413	74.00	Current	At least once per month or more on 5-point scale (daily, every week, every month, less than monthly, at an earlier age)	7.20
Petit et al [61, 62]	Canada	Longitudinal	2–6 13	14921011	55.77 37.79	12 months	‘sometimes’ or ‘frequently’ response on 4-point scale (never, seldom, sometimes, frequently)	14.50 12.80
Simonds & Parraga [63]	USA	Cross-sectional	5–18	309	83.74	6 months	At least one episode	10.03
Smedje et al [64, 65]	Sweden	Longitudinal	5–7 6–8	1844635	83.25 34.44	6 months	Any response greater than ‘never’ on 5-point scale (never, occasionally, once or twice per week, 3 or 4 days per week, at least 5 days per week)	8.30
Stallman et al [66]	Australia	Cross-sectional	5–10	1814	25.2	1 week	at least once; 4-point scale (never, rarely, sometimes, usually	10.5
Steinsbekk et al [67, 68]	Norway	Longitudinal	4	995	79.60	3 months	Anders criteria[69]	0.70
			6	795	63.60			3.50
Tomás Vila et al [70]	Spain	Cross-sectional	6–17	887	68.75	Lifetime	Yes/no	12.50
Vaher et al [71]	Estonia	Cross-sectional	8–9	703	66.00	Current6 months	Yes/no	28.59
Wiechers et al [31]	Germany	Cross-sectional	*M* = 9.6	1144	65.00	Current	‘sometimes’ or ‘often’ on 3-point scale (never/rarely, sometimes, often)	9.20
Xiong et al [72]	China	Cross-sectional	^b^	2848	^b^	^b^	^b^	2.84
**Diagnosis**								
Fisher et al [73]	UK	Longitudinal	12	6796	46.23	6 months	DSM-IV	12.55
Ozgun, et al [74]	Turkey	Cross-sectional	6–18	4144	83.7	Current	ICSD-2	4.20
Ramírez et al [75]	Columbia	Cross-sectional	5–12	296	91.60	ns	DSM-IV	7.40
Shang et al [76]	Taiwan	Cross-sectional	4–9	1391	91.60	Lifetime	Yes/no DSM-IV	8.60
						1 month		1.00

ns = not specified

^a^exact data reported in a second paper is not included in this paper

^b^full paper not accessible

**Table 2:** Characteristics of Included Studies of Adults listed by Measurement Type.

Citation	Country	Design	Age	*N*	Response Rate %	Prevalence Outcomes		Results %
**Self-reported behaviour**								
Bixler et al [77]	USA	Cross-sectional	18–80	1006	ns	Lifetime	Yes/no	2.5
						Current		0.4
Bjorvatn et al [78]	Norway	Cross-sectional	18–96	1000	25.38	Lifetime	At least once during past 3 months from 6-point scale (never, less than once per month, less than once per week, 1–2 days per week, 3–5 days per week, daily/almost daily)	22.40
						3 month		1.70
Davis [79]	England	Cross-sectional	ns	100	ns	Lifetime	Yes/no	3.0
Hirotsu et al [80]	Brazil	Cross-sectional	16–60+	2017	99.95	Current	Yes/no	1.00
Mume [11]	Nigeria	Cross-sectional	18–60	228	91.20	Lifetime	Yes/no	7.00
Orme [81]	UK	Cross-sectional	ns	151	ns	Lifetime	Yes/no	13.91
Panda et al.[82]	India	Cross-sectional	16–55	1050	>95	1 month	ns	.60
Stepansky et al [83]	Austria	Cross-sectional	ns	1000	ns	ns	ns	.004
Thomas & Pederson [84]	USA	Cross-sectional	19–34+	1116	88.35	Current	ns	2.24
Vela-Bueno et al [85]	Spain	Cross-sectional	18–65+	1131	75.40	Lifetime	Yes/no	1.10
Zeitlhofer et al (2010)	Austria	Cross-sectional	14 = 50+	1000	82.10	Current	ns	2.00
**Diagnosis**								
Frauscher et al [86]	Austria	Cross-sectional	19–77	100	27.50	Lifetime	non-bothersome sleepwalking < 2 times per week ICSD-2	12
Oluwole [87]	Nigeria	Cross-sectional	19–35	276	30.84	Lifetime	Yes/no ICSD	4.35
						2 weeks		1.45
Ohayon [88]	UK	Cross-sectional	15–100	4972	79.60	Current	DSM-IV	2.00
Ohayon et al [89]	USA	Cross-sectional	18–102	15 929	83.2	12 months	DSM-IV	3.6
						Childhood		25.7

#### Risk of bias

The risk of bias analyses are presented in Table 3. There were no studies without risk of bias and all failed to control for bias across multiple criterion.

**Table 3:** Summary of Risk of Bias in Included Studies.

	Target Population	Probability sampling	Selection bias	Standardized data collection	Measurement Reliability	Measurement Validity	Analyses accounts for sampling design	Period Assessed	95% CI
Abdel-Khalek (2001)	1	1	1	0	1	1	1	Current	8.9–11.2
Agargun et al (2004)	0	0	2	0	1	1	1	Current	0.6–2.0
Bharti et al (2006)	0	1	2	0	1	1	1	Current	0.5–7.4
Bixler et al (1979)	0	0	2	0	1	1	1	Current	0.2–1.1
								Lifetime	1.7–3.7
Bjorvatn et al (2010)	0	0	0	0	1	1	0	Current	4.7–9.2
								Lifetime	19.9–25.1
Blader et al (1997)	0	2	1	0	1	1	1	Current	8.1–11.8
Cai et al (2008)	0	2	2	2	1	1	2	Current	0.6–1.3
Fisher & Wilson (1987)	0	0	1	1	1	1	1	Current	19.1–23.0
Fisher et al (2014)	0	0	1	1	1	1	1	Current	11.8–13.4
Furet et al (2011)	0	2	2	2	1	1	2	Current	0.06–3.3
Ghalebandi et al (2011)	0	0	0	0	1	1	0	Current	3.4–4.5
Goodwin et al (2012)	0	2	2	2	1	1	2	Current	2.2–5.6
Hirotsu (2014)	0	0	1	0	1	1	0	Current	.06–1.5
Kilincaslan et al (2014)	0	0	1	2	1	1	0	Current	5.0–6.5
Lehmkuhl et al (2008)	0	0	1	0	1	1	1	Current	2.8–3.8
Lui (2003)	0	0	0	2	1	1	0	Current	4.3–8.4
Lui (2005)	0	0	0	0	1	1	0	Current	0.4–0.8
Mume (2010)	0	0	0	2	1	1	1	Current	6.7–7.3
Neveus et al (2001)	0	0	0	2	1	1	1	Current	6.1–8.4
Ohayon et al (1999)	0	0	0	0	1	1	0	Current	1.6–2.4
Ohayon et al (2012)	0	0	0	0	1	1	0	Current	3.3–3.9
								Lifetime	25.0–26.4
Ozgun et al (2013)	0	2	0	2	1	1	1	Current	3.6–4.9
Panda (2012)	0	1	2	0	1	1	1	Current	0.3–1.3
Ramírez et al (2008)	0	2	2	2	1	1	2	Current	4.9–11.0
Shang et al (2006)	0	0	1	0	1	1	1	Current	.06–1.7
								Lifetime	7.2–10.2
Simonds & Parraga (1982)	0	2	1	2	1	1	1	Current	7.6–14.6
Smedje et al (1999; 2001)	0	0	1	2	1	1	1	Current	7.1–9.6
Stallman et al (2016b)	0	0	2	0	1	1	2	Current	9.2–12.0
Stallman et al (2016a)	0	0	0	0	1	1	1	Current	1.8–4.7
Steinsbekk et al (2013, 2015)	0	1	1	0	1	1	0	Current	0.3–1.5
								Current	2.4–5.0
Stepansky et al (1999)	0	0	2	2	1	1	2	ns	0–100
Thomas & Pederson (1963)	0	1	0	0	1	1	1	Current	1.6–3.4
Tomás Vila et al (2008)	0	0	1	2	1	1	2	Current	10.5–14.8
Vaher et al (2013)	1	2	2	0	1	1	1	Current	25.4–32.0
Wiechers et al (2011)	0	0	0	0	1	1	0	Current	7.7–11.0
Xiong et al (2008)	0	0	2	2	1	1	2	Current	2.3–3.5
Yang et al (1987)	0	1	2	2	1	1	1	Current	2.9–5.6
Zeitlhofer et al (2010)	0	0	0	0	1	1	0	Current	7.5–8.0
Abe (1966)	0	0	2	0	1	1	1	Lifetime	2.8–6
Abe et al (1982)	1	1	1	0	1	1	1	Lifetime	2.9–7.3
Archbold et al (2002)	1	1	0	0	1	1	1	Lifetime	12.8–17.1
Buhler (1981)	0	0	0	0	1	1	0	Lifetime	3.1–8.0
Davis (1942)	1	1	2	2	1	1	1	Lifetime	1.0–8.9
Frauscher et al (2014)	0	0	2	0	1	1	2	Lifetime	35.1–36.9
Greene et al (2015)	0	1	1	0	1	1	1	Lifetime	22.3–25.2
Ipsiroglu et al (2002)	0	1	2	0	1	1	0	Lifetime	11.6–19.4
Klackenberg (1971, 1982, 1987)	0	2	2	1	1	1	1	Lifetime	33.1–47.3
Laberge et al (2000)	0	0	1	2	1	1	1	Lifetime	4.7–7.2
Oluwole (2010)	0	1	2	2	1	1	1	Lifetime	1.1–5.5
Orme (1967)	1	1	2	2	1	1	1	Lifetime	9.2–20.4
Petit et al (2007, 2015)	0	0	1	0	1	1	0	Lifetime	12.8–16.4
Vela-Bueno et al (1999)	2	2	1	2	1	1	2	Lifetime	0.6–1.9

Note. 0 = no risk of bias

1 = risk of bias

2 = unclear risk of bias

ns = not specified

#### Sampling

Criterion 1—Target population clearly defined: The majority of studies defined their target population (n = 44), six had risk of bias and one was unclear. Criterion 2 -Probability sampling used to identify potential respondents: More than half of the studies used probability sampling (n = 30), 12 had clear risk of bias, and 9 had an unclear risk. Criterion 3—Representativeness of the sample: There was significant heterogeneity in regards to how representative the samples were. For studies that reported or provided data to calculate response rates (n = 44), they ranged from 4.96% to 99.95%. Twenty-three studies met the minimum criteria of 70% response rate to be considered representative of the population [21]. Overall, only 15 papers had no risk of bias for sampling, 17 had risk of bias and 19 had unclear risk.

#### Measurement

Criterion 4—Standardized data collection procedures: More than half of the studies had no risk of bias related to data collection procedures (*n =* 29), three had risk of bias, 19 had unclear risk. Criterion 5—Reliable measurement: No studies reported the reliability of the measure they used to assess sleepwalking. Criterion 6—Valid measurement: No measures of sleepwalking used in any study have been validated. Measurement of sleepwalking varied considerably between studies. All studies used interview or questionnaires to measure sleepwalking—no studies included objective measures. Questions ranged from a single question to diagnostic interviews. Studies used either self-reported behavior (*n* = 15), or parent report of behavior (*n* = 27), self-report to form a diagnosis (*n* = 4), or parent-report diagnosis (*n* = 4), One study used more than one assessment measure of sleepwalking, collecting both parent and child reports [31]. Three studies encouraged participants to consult with a parent or family member, but did not assess or report the extent that was done [11, 32, 33].

#### Analyses

Criterion 7—Sampling design accounted for in the analysis: Thirteen studies has no risk of bias, 28 had risk of bias and 10 had unclear risk. Criterion 8—Confidence intervals for statistical estimates: The confidence intervals were calculated for each study and are summarized in Table 3.

As a result of the significant risk of bias in all studies and the great variations between studies including definitions of sleepwalking, data collection, measuring and reporting of results, a series of a priori decisions were made with respect to combining data. All studies were included and no sensitivity analyses were able to be conducted. For lifetime prevalence, the highest rate reported in a study was used as the lifetime prevalence rate. As the true lifetime prevalence rate cannot decrease over time, differences at different time points in the one study are likely to reflect attrition and/or recall bias. Period prevalence rates varied considerably between studies—previous week, previous one month, 3 months, 6 months, 12 months, or period not specified. The small sample size for any given period and the significant risk of bias within studies make it problematic to ascertain the prevalence at each point. Consequently the studies that reported period prevalence rates between one week and 12 months were combined to provide an estimate of the current prevalence of sleepwalking, defined here as sleepwalking within the past year. Where studies reported multiple time points within the previous 12 months, the highest rate is taken to reflect the greatest proportion of participants sleepwalking within the previous 12 months. Where multiple papers for a single study reported the current prevalence rate at different ages, all that did not overlap in the age range were included in the calculation of current prevalence rate. When studies reported that parents and children completed interviews or questionnaires together, these were recorded as parent-reported prevalence rates.

### Current prevalence of sleepwalking

**Fig 2:**
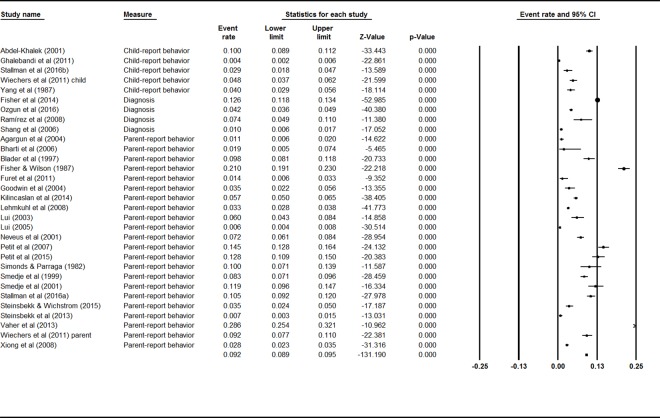
Forest plot for Current Sleepwalking in Children.

Mixed effects analysis showed a significant difference between the current prevalence rates for adult and child samples, *Q* = 22.25, *p* < .001, indicating the need to consider these populations separately. Thirty-one studies reported rates for various periods to provide current prevalence rates for sleepwalking behavior in children. A mixed effects analysis showed no significant difference between studies using child self-report behavior, parent-report behavior, and sleepwalking diagnoses, *Q* = 1.49, *p* = .48, indicating that they could be analyzed together. The event rates and 95% confidence interval across ages are shown in Fig 2. The combined effect for the current prevalence of sleepwalking during childhood was 5.0% (95% CI 3.8–6.5). There was high and significant heterogeneity between studies (*I*^2^ = 98%; *Q* value = 969.45, *p <* .001), indicating great variability in effect size estimates.

**Fig 3:**
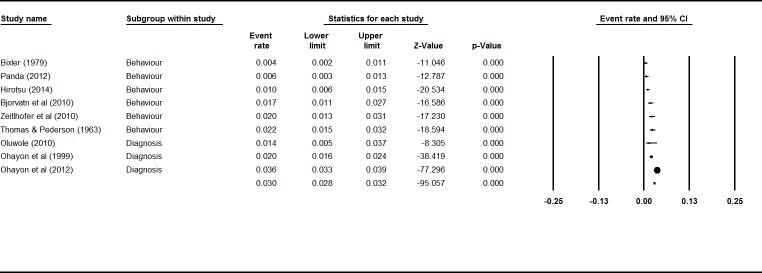
Forest plot for Current Sleepwalking in Adults.

A mixed effects analysis showed no significant difference between self-reported sleepwalking behavior and diagnoses in adult studies (*Q* = 3.57, *p* = .06). The event rates and 95% confidence interval across ages are shown in Fig 3. The combined effect for the current prevalence rate of sleepwalking in adults from nine studies was 1.5% (95% CI 1.0%–2.3%). There was high and significant heterogeneity between studies (*I*^2^ = 93%; *Q* value = 108.65, *p* < .001), indicating great variability in effect size estimates.

### Lifetime prevalence of sleepwalking

**Fig 4:**
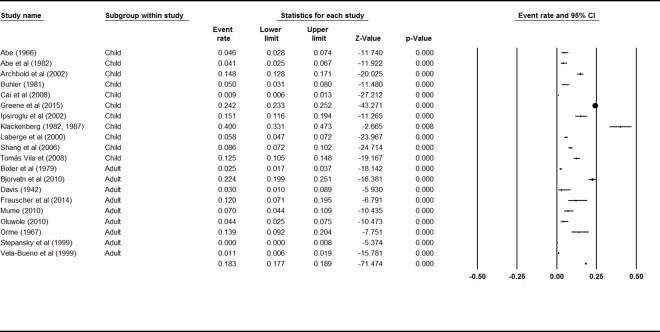
Forest plot for Lifetime Sleepwalking.

There was no significant difference for lifetime prevalence rates of sleepwalking between adults and children, *Q* = 1.65 *p* = .20, indicating that the 20 studies could be analyzed together. The event rates and 95% confidence interval across ages are shown in Fig 4. The combined effect for lifetime prevalence of sleepwalking was 6.9% (95% CI 4.6%–10.3%). There was high and significant heterogeneity between studies (*I*^2^ = 98%; *Q* value = 1238.95, *p* < .001), indicating great variability in effect size estimates.

### Developmental trends in sleepwalking

**Fig 5:**
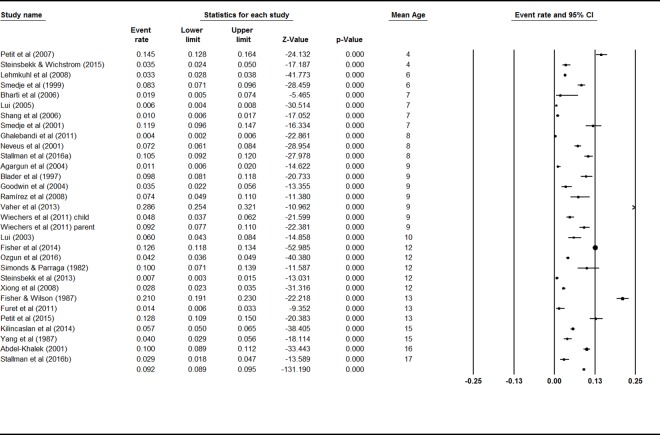
Forest Plot for child current sleepwalking prevalence ordered by mean sample age.

Pediatric studies of sleepwalking in childhood included children aged between two and 18 years. The ages varied considerably between studies. The reporting of prevalence rates was diverse and included prevalence at a given age, prevalence within the sample’s age range and only reporting the mean age of the sample and a prevalence rate. In order to assess the developmental trend of sleepwalking across childhood, the mean age for each sample was calculated for each study. The event rates and 95% confidence interval across ages are shown in Fig 5. Meta-regression showed no significant relationship between the mean age of children reported in studies and the current prevalence rate of sleepwalking (*Q* value = 0.34, *p* = .56, *R*^2^ = 0). There was high and significant heterogeneity between studies (*I*^2^ = 98%; *Q* value = 1943, *p* < .001) indicating great variability in effect size estimates.

### Bias and heterogeneity

Inspection of the funnel plots showed significant bias with positive skew for both current and lifetime prevalence rates. The Egger’s test was significant for bias for current child (*t* (29) = -8.66, *p* < .001) and adult analyses (*t* (7) = -4.36, *p* < .001) and lifetime analyses (*t* (18) = -7.99, *p* < .001). The Classic fail-safe *N* shows no evidence of publication bias with 4247 missing lifetime studies needed to bring *p*-value to non-significance, 8 010 adult current and 8 707 child current studies.

## Discussion

This systematic review included more than 100 000 people from 51 studies to identify the prevalence rate of sleepwalking in adults and children. Sleepwalking has been reported in children as young as two years and throughout adulthood. The lifetime prevalence for sleepwalking was 6.9% (95% CI 4.6%–10.3%). This does not vary significantly between childhood and adulthood, suggesting that relatively few people start sleepwalking later in life. This is consistent with adult onset of sleepwalking being associated with medications [20] and neurodegenerative diseases (e.g.[34]). This finding highlights the importance for detailed clinical evaluations of patients presenting with sleepwalking for the first time in adulthood. The current rate of sleepwalking was higher in children than adults 5.0% (95% CI 3.8–6.5) than in adults 1.5% (95% CI 1.0%– 2.3%). This difference may be the result of methodological issues or the decrease in slow wave sleep evident between childhood and adulthood [35].

The strengths of this study are its large sample size, both for children and adults, and the large number of studies included in the analyses. The limitations relate to the methodological problems within studies that are reflected in the high risk of bias across all studies and the consequent high heterogeneity across all analyses. Sleepwalking research is hampered by the very nature of the phenomenon. It occurs at night while the individual is sleeping. No studies included in this review used objective measures of sleepwalking; all relying on self- or parent-reports of sleepwalking behavior. Sleepwalkers typically have poor memory of sleepwalking episodes, because like other NREM dreams, sleepwalking actions appear less bizarre and novel than REM dreams [36]. The identification of sleepwalking episodes is therefore heavily dependent on the behavior being observed by others or the sleepwalker suspecting it because of injuries or other occurrences, such as noticing that things in the house have been moved, when they awaken. This is supported by research that included relationship status, showing that sleepwalking is more prevalent in married people than single people [11]. It may also mean that the significant difference in current sleepwalking rates between children and adults is an artifact of not being observed, rather than a true effect. It is important that future research measure who participants live with and how they or the informant know the person has been sleepwalking, and then control for these factors when determining prevalence rates. When using parent-report in older children, it would be important to ascertain whether parents are awake and able to observe adolescents sleepwalking in order to better determine the validity of the measure.

Reliability and validity have not been evaluated for self-, or parent-report measures of sleepwalking. This likely accounts for the very high heterogeneity in the results of this study. Because the definition of sleepwalking varied considerably across studies, random effects modelling was used in this study as it does not assume one underlying true effect across measures. In contrast, clinical diagnostic measures, such as the DSM, have diagnostic criteria that include recurrent episodes, observed sleepwalking behavior with impaired functioning [9]. Prevalence rates would have therefore been expected to be lower than for sleepwalking behavior. However, this was not evident in the current study, likely due to the enormous heterogeneity in the sample. It is recommended that single behaviors, in addition to frequency or recurrent sleepwalking be assessed in future epidemiological studies to overcome this problem. The presence of impairment or distress is a consequence of sleepwalking behavior and should not be used to calculate prevalence rates. Few studies that assessed current period prevalence of sleepwalking, assessed incidence. This is an important component for future studies to include in order to be able to understand the developmental trajectory of sleepwalking.

There was also inconsistency across studies in recall periods. It would be sensible for future research to use the last 12 months to capture current sleepwalking, in addition to a recent period—such as previous two weeks—that is less affected by recall bias. Previous year period prevalence and incidence measurement would also readily enable the developmental trajectory of sleepwalking to be better observed. Other periods such as one, three and six months do not add anything of importance to our understanding of sleepwalking.

## Conclusion

Prevalence rates of sleepwalking are challenging to measure due to methodological limitations in identifying and accurately measuring the behavior. These include: a) the event happening during sleep when an individual is least likely to be witnessed; b) the individual having little or no memory for the event; c) reliance on recall of retrospective events by the sleepwalker or the informant; and d) differences in the conceptual understanding and measurement of the behavior. Any estimates, therefore, are likely to underestimate the true prevalence rates and represent episodes that are known to either caregivers (in relation to child studies) or the participant (in the case of adult studies). The summary prevalence rates reported here have significant limitations associated with systematic measurement error and should be used cautiously. The combined child and adult data supports the notion that sleepwalking behavior is a relatively common occurrence sometime during the lifespan. This review highlights the need for further epidemiological research to accurately explore the prevalence and incidence of sleepwalking across the lifespan. Outcomes would be strengthened by methodologies that: a) concurrently assess sleepwalking behavior and sleepwalking disorders; b) triangulate results using subjective and objective measures; c) assess how individuals know they sleepwalk; and d) assess injuries to sleepwalker and others.

## Supporting Information

S1 Table PRISMA Checklist. (DOC)
